# Systematic review of modifiable risk factors shows little evidential support for most current practices in *Cryptosporidium* management in bovine calves

**DOI:** 10.1007/s00436-020-06890-2

**Published:** 2020-09-30

**Authors:** Julii Brainard, Lee Hooper, Savannah McFarlane, Charlotte C. Hammer, Paul R. Hunter, Kevin Tyler

**Affiliations:** Norwich Medical School, University of East Anglia, Norwich, NR4 7TJ UK

**Keywords:** Calves, Cryptosporidiosis, Risk factors, Colostrum, Organic, Herd size, Flooring, Co-infection

## Abstract

Cryptosporidiosis is common in young calves, causing diarrhoea, delayed growth, poor condition and excess mortality. No vaccine or cure exists, although symptomatic onset may be delayed with some chemoprophylactics. Other response and management strategies have focused on nutritional status, cleanliness and biosecurity. We undertook a systematic review of observational studies to identify risk or protective factors that could prevent *Cryptosporidium parvum* infection in calves. Included studies used multivariate analysis within cohort, cross-sectional or case-control designs, of risk factors among young calves, assessing *C. parvum* specifically. We tabulated data on characteristics and study quality and present narrative synthesis. Fourteen eligible studies were found; three of which were higher quality. The most consistent evidence suggested that risk of *C. parvum* infection increased when calves had more contact with other calves, were in larger herds or in organic production. Hard flooring reduced risk of infection and calves tended to have more cryptosporidiosis during warm and wet weather. While many other factors were not found to be associated with *C. parvum* infection, analyses were usually badly underpowered, due to clustering of management factors. Trials are needed to assess effects of manipulating calf contact, herd size, organic methods, hard flooring and temperature. Other factors need to be assessed in larger observational studies with improved disaggregation of potential risk factors.

**Electronic supplementary material:**

The online version of this article (10.1007/s00436-020-06890-2) contains supplementary material, which is available to authorized users.

## Background

*Cryptosporidium parvum* is a common protozoan parasite in cattle. It causes chronic diarrhoea (scour) leading to stunted growth, loss of yield and potentially death (Thomson et al. 2017; Wells and Thomson 2014). Young calves (under 6 weeks old) are at greatest risk of both catching and spreading pathogenic infection (Silverlås et al. 2009a; Wells and Thomson 2014). Economic costs in Great Britain were estimated in 2014 to be £100–£200 per infected calf (Shaw 2014), arising mostly from veterinary treatment, reduced future milk yield and lower weight gain. Prevalence of *C. parvum* in stool samples of European cattle herds were reported to range from 13 to 100% (Imre and Dărăbus 2011). Cattle are recognized as an especially important reservoir for *C. parvum*, which can spread from cattle to other animals or to humans through many routes (Brankston et al. 2018; Hunter and Thompson 2005; Wells and Thomson 2014). Globally, infection from *C. parvum* and other *Cryptosporidium* species (e.g. *hominis*) are important contributors to total human deaths from diarrhoeal illness (Vermeulen et al. 2017). Large outbreaks in humans (affecting dozens or even hundreds of people) from pathogenic *C. parvum* infection regularly occur in Europe (Cacciò and Chalmers 2016).

Control of *C. parvum* is therefore highly desirable for good animal welfare, to reduce risks to human health and to limit economic losses in affected industries. An evidence review (Wells and Thomson 2014) reiterated that treatment options are limited; for instance, in the UK only two products are licenced to treat cryptosporidiosis in calves (halofuginone lactate marketed as Halocur®) and paromomycin sulphate. Halocur® treatment is more common and appears to delay peak shedding rather than cure disease while it is toxic at a dose close to that of efficacy.

We undertook a systematic review was to inventory management risk factors related to *C. parvum* infection in very young cattle. A systematic review research design is intended to produce an unbiased summary of available evidence using comprehensive search and synthesis strategies (Deeks et al. 2011). An a priori hypothesis about which risk factors were believed previously to be important was not specified, required or appropriate to the objectives of this systematic review. Such a systematic review on risk factors for cryptosporidiosis in bovine calves has not been produced previously, although there are published articles in other review designs that addressed risk factors for multiple pathogen causes of calf diarrhoea, including but not specific to *C. parvum* (Cho and Yoon 2014; Muktar et al. 2015).

## Methods

PRISMA systematic review reporting guidelines were followed (Toews 2017).

### Population

Eligible studies had to address infection in bovine calves (*Bos Taurus*) under 4 months old. The vast majority of calves suffering from cryptosporidiosis are under 1 month old (Erbe 2010; Wells and Thomson 2014). Older livestock are also managed differently from very young animals, so they may experience different environments and risk transmission pathways. Articles on humans, related species such as buffalo or yaks, and other animals were ineligible. Studies on hybrids of cattle with other animals (e.g. beefalo) or mixed species herds (of *Bos Taurus* mixed with others) were considered individually, in case they provided sufficient cattle-specific information to be informative.

### Exposure

Selected studies had to address potentially modifiable risk factors related to *C. parvum* oocyst shedding. To be eligible, studies had to include some adjustment for potential confounders. This could include multivariate risk factors from modelling or other adjustment for at least two risk factors: studies with only univariate model results were not eligible.

### Outcome

The outcome was *C. parvum* oocyst shedding. Evidence that other *Cryptosporidium* species are likely to be pathogenic in bovine calves is almost non-existent (Thomson et al. 2017; Wells and Thomson 2014), yet cows very often carry other species of *Cryptosporidium*. Studies were only eligible if *C. parvum* infection was confirmed by (A) molecular methods (ELISA, rtPCR), (B) immunofluorescence microscopy or (C) contrast microscopy that detected oocysts that was concurrent with a large percentage of symptomatic animals (≥ 90% with diarrhoea).

### Study designs and language

Any concurrent observational design (cohort, cross-sectional or case-control but not pre−/post periods) was eligible. Studies were excluded if not available in a language known to the authors (English, German, Spanish or French) or if the article could not be easily translated into English using Google Translate. Articles without abstracts or available full text were excluded.

### Search strategy

We searched these databases from inception to May/June 2019: Scopus, CAB International abstracts, MEDLINE (PubMed) and Embase. A limited grey literature search was undertaken of three government databases via websites in summer 2019: The UK Dept for Food and Rural Affairs, The US Dept. of Agriculture library (at Cornell University) and The European Commission, Agricultural and Rural Development section. Conference proceedings were not searched. Literature databases were chosen following recommendations about the most comprehensive bibliographic sources for veterinary science research (Grindlay et al. 2012).

The search terms were designed to make sure they found relevant articles but with a minimum of extraneous (irrelevant search return) results. Grey literature search terms were *Cryptosporidium*, cryptosprodiosis and *parvum*. Forward and backward citation searches of included articles were not done to look for additional studies. Within the peer-review bibliographic databases, we searched for, among title/abstract/keywords:

At least one of (*Cryptosporidum*, *C. parvum*, cryptosporidiosis).

AND

At least one of (calf, cattle, cow, bull, dam, dairy, beef, herd, calves).

### Study selection and data extraction

After de-duplication, titles and abstracts were independently screened by two investigators (JB and CCH) against the inclusion criteria. Items were chosen for full text review or excluded. Selection disagreements were resolved by discussion or on the verdict of a third reviewer (PRH). Full texts were obtained where possible. Decisions about final inclusion or exclusion were made after full text review by one or more authors. Full-text review and data extraction were undertaken by LH, SM or JB and checked by each other.

Any risk or protective factors reported to be statistically significant at a *p* ≤ 0.05 level of confidence were extracted and included in the results. After all such significant factors were identified and extracted, we checked back within the included studies to find instances where each factor had been assessed but found not to be a significant risk factor (to assess the consistency of importance of each factor and to reduce the risk of being influenced by random findings).

### Quality assessment

Quality assessment was undertaken by LH or SM and checked by JB. Modified questions from the CASP checklist for cohort studies (Critical Appraisal Skills Programme 2017) were used to generate a customized quality assessment with constant decision criteria, using a data extraction and quality assessment form developed for this review (Supplementary file 1). Four quality categories were identified from the assessment exercise, as detailed in the Supplementary file.

### Reporting and synthesis

Characteristics of included studies were tabulated and we carried out narrative synthesis, grouping by risk factor categories. Meta-analysis was not attempted because of the diversity of ways that exposures were reported.

## Results

**Fig. 1:**
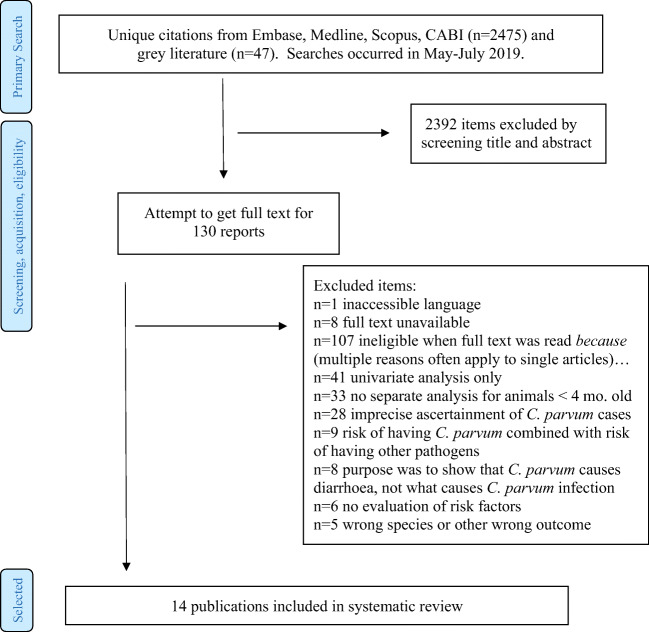
Study selection procedure

Our search found 2522 possible relevant studies, see Fig. 1. From screening abstracts and titles, 130 of these appeared to potentially be risk assessments. Eight full texts were unavailable, and one was available but written in a language we could not read or translate (Persian: Changizi et al. 2012). We eliminated 107 articles because they did not contain information specifically about *C. parvum* or the only risk factor assessed was age, or the only risk factor information was unadjusted (they considered risk factors only individually, never in combination with each other). Fourteen studies were eligible for inclusion and were data extracted and quality assessed. Characteristics of the included studies are found in Table 1 and quality assessment results in Table 2.

**Table 1:** Characteristics of scientific studies included in this review

Study	No. of animals	No. of indiv. herds	Herd location(s)	Detection methods	Ages of sampled calves	Type of production	Prevalence of C. parvum infection
Al Mawly et al. (2015b)	1283	97	New Zealand	Immunofluorescence and PCR	1–5 or 9–21 days old	Dairy	5.8% 1–5 days old; 16% when 9–21 days old
Brook et al. (2008)	Max. 215	41	UK (NW England)	Stained microscopy confirmed by PCR	< 100 days, median ~ 26 days	Mix	28% of samples
Díaz et al. (2018)	Max. 147	22	Italy	Microscopy and PCR	< 35 days	Dairy	38.8% of samples
Imre et al. (2015)	428	20	Romania	ELISA	1–30 days	Mix	37.4% overall; peak 49.3% at 8–14 days old
Maddox-Hyttel et al. (2006)	377	Up to 50	Denmark	Epifluorescence microscopy	< 1 month old	Dairy	98%
Matoock et al. (2005)	106	1	Egypt	Microscopy and ELISA	< 8 weeks old	Dairy	10.6% overall; 4.4% at 7–8 weeks to 26.5% 2–4 weeks
Silverlås et al. (2009b)	500	50	Sweden	Epifluorescence microscopy	≤ 2 months old	Dairy	96% of herds; 0–71% within herds
Sischo et al. (2000)	486	11	USA, New England	Immunofluorescence microscopy	≤3 months old	Dairy	2% (4–8 weeks old) to 15% (0–3 weeks)
Starkey et al. (2005)	Unclear (hundreds)	39	USA, NY	Contrast microscopy and ELISA	Newborn	Dairy	Unclear
Szonyi et al. (2012)	391	44	USA, NY	PCR RNA	< 65 days old	Dairy	59.1% < 1 month old, 5.2% 1–2 months old
Trotz-Williams et al. (2007)	1045	11	Ontario, Canada	PCR	< 30 days old	Dairy	78% of all calves samples at least 4 times
Trotz-Williams et al. (2008)	1089	119	Ontario, Canada	PCR	7–28 days	Dairy	30%, 0–80% within individual herds
Urie et al. (2018)	2249	104	USA (coast to coast)	Immunofluorescence microscopy	3–66 days (mean 22 days)	Dairy	43.10%
Weber et al. (2016)	63	20	Switzerland	ELISA	Up to 6 weeks old	Mix, mostly dairy	~ 50%

**Table 2:**
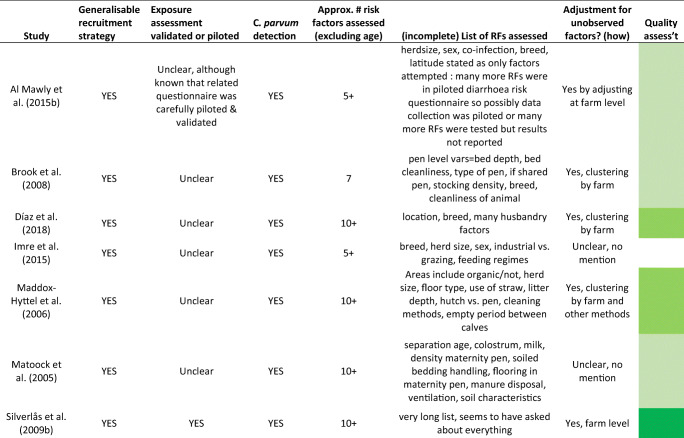

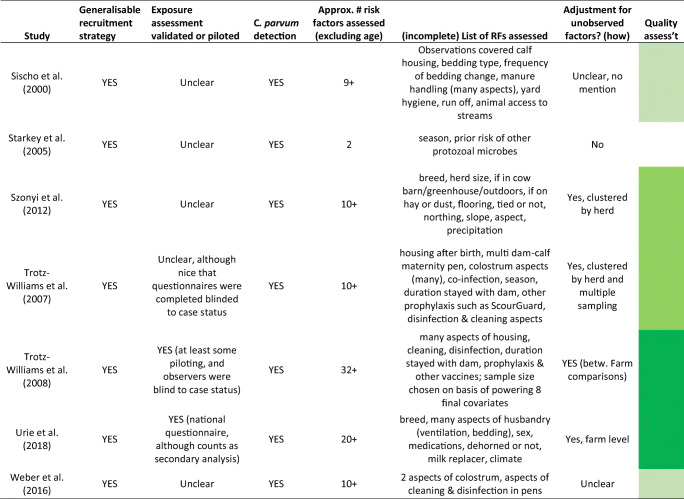
Quality assessment for studies included in this literature review

Explanation is provided for how quality questions were answered in Supplementary file 1. Some studies reported the total number of samples rather than total number of animals sampled; most of these reports implied that the same animal was not meant to be sampled more than once. Key to quality assessment colours: darkest green = most reliable, the lighter the green the more uncertainty and less confidence in the study findings, clear = lowest quality study

The calves were overwhelmingly part of dairy production (11 studies included only dairy, three were mixed dairy and beef). Studies were carried out in Europe (6 studies), North America (6 studies), New Zealand and Egypt (one study each). Prevalence of *C. parvum* was 6–78% of individual calves within studies, and studies assessed 1–119 herds and 63 to 2249 individual animals. As management interventions tend to differ by herd, rather than by animal, the studies were all limited in their ability to identify important risk factors.

We found that three studies were the strongest methodologically: Trotz-Williams et al. (2008) carried out in 119 Canadian herds, sampling 1089 calves of 7–28 days old; Urie et al. (2018) carried out in 104 US herds, sampling 2249 calves aged 3–66 days; and Silverlas and colleagues (2009) carried out in 50 Swedish herds including 500 calves aged up to 2 months (Silverlås et al. 2009b; Trotz-Williams et al. 2008; Urie et al. 2018). The observations from these three studies are reported separately in the narrative summaries.

### Risk factors tested but not found to be significant in any adjusted models

Many risk factors were tested and found to be unimportant. Lower quality studies tested but did not find these items to be significant risk or protective factors (*n* = the number of studies that considered this potential risk factor): sex of animal (*n* = 1), cleanliness of actual animal (*n* = 1), breeding system (*n* = 1), dairy or beef farming (*n* = 1) and access to stream as water supply or not (*n* = 1: Sischo et al. 2000). Among the three higher quality studies: Silverlås et al. (2009b) found that calf age at weaning was not a risk factor. Urie et al. (2018) did not link risk of infection to any of these factors: birth weight, average daily weight gain, protein intake, fat intake, whether dam was multiparous, whether the birth was assisted, whether the birth was single or twins, whether the calf’s navel was disinfected, sex of primary caretaker and whether calves were dehorned. Urie et al. (2018) and Trotz-Williams et al. (2008) observed and reported on many feeding aspects that were not significant in their final adjusted models: bacterial count in liquid feeds, use of a pasteurized liquid diet, supplying calf starter at <7 days old, bottle-bucket-bar or other types of feed supply mechanisms.

### Birth management

#### Time dam spent in maternity pen(s) away from the main herd

Two studies assessed use of maternity pens, isolating dams from the herd in a period prior to giving birth. A moderate quality study (Maddox-Hyttel et al. 2006) found that this segregation had no effect on infection risk, but a higher quality study (Silverlås et al. 2009b) found that longer time spent in maternity pens had a protective effect against infection. In Silverlås et al., the odds ratio for calves developing *C. parvum* became as low as 0.12 (95%CI 0.02 to 0.7) when dams were in the maternity pen >3 weeks prior to birth, compared with when dams were only in the maternity pen for ≤2 days before birth. There is some higher quality but limited evidence that longer segregation of dams from the rest of the herd prior to birth is protective.

#### Shared maternal pens

When a calf was born into a pen that held multiple dams (rather than single dam), this increased risk of disease in Matoock et al. (2005) (OR 5.2, no variance reported), but was not found to increase disease risk in Trotz-Williams et al. (2007) (OR 0.96, 95%CI 0.69–1.33) or Weber et al. (2016). No higher quality study assessed this risk. Evidence is limited and mixed about whether dams sharing birthing pens matters.

#### Time calf spent after birth with dam

It seems likely that keeping calves with dams for long after birth was rarely assessed because in the dairy industry separation of calves from dams very soon after birth is standard practice. Just two studies, both higher quality (Silverlås et al. 2009b; Trotz-Williams et al. 2008) considered this as a management risk factor. Separation at birth had no impact in Trotz-Williams et al. (2008), but longer stays with the dam (up to 4 days) were increasingly protective in Silverlås et al. (2009b). Compared with separation ‘soon’ after birth, staying with the dam up to 4 days reduced risk of *C. parvum* infection OR to 0.11 (95%CI 0.02–0.52) in Silverlås et al. (2009b). Trotz-Williams et al. (2007) also considered a relevant outcome, risk of diarrhoea, and found that this was higher (OR 1.58, 95%CI 1.34–1.86) for calves who stayed >1 h with the dam after birth. The evidence about length of time spent with dam after birth is inconclusive.

### Breed

Six studies considered whether certain breeds were more vulnerable to infection, of which one study (Imre et al. 2015) found that ‘pure-bred’ animals (pure versus cross breed not defined) were at higher risk than cross breeds. Three studies (Maddox-Hyttel et al. 2006; Szonyi et al. 2012; Urie et al. 2018) assessed Holstein vs. Jersey calves and found no breed-related risks in multivariate analyses. Two other studies (Al Mawly et al. 2015b; Brook et al. 2008) looked for other breed differences (other breeds were compared, not just Holstein vs. Jersey) and found no differences. Urie et al. (2018) is the only higher quality study that definitely considered breed as a risk factor (Holsteins vs. Jerseys) and found that it did not matter. There is no clear support for significant differences in susceptibility to *C. parvum* between the major breeds of dairy cattle.

### Colostrum

Colostrum intake is known to be important to establishing healthy immune systems in bovine calf neonates (Strekozov et al. 2008). An important difficulty with assessing whether colostrum intake has an impact on subsequent *C. parvum* disease is that it is not consistently reported whether calves had colostrum or what processing the colostrum had or how it was delivered (whether it was heat treated, from pooled tanks or hand fed). We further suspect some studies reported that calves had ‘colostrum’ when in reality calves had artificial colostrum. Some studies report universal or near-universal exposure status (e.g. all calves had similar colostrum doses or no colostrum), so colostrum feeding could not be assessed as a risk factor. Colostrum that has been sterilized or stored loses antibody effectiveness (Elizondo-Salazar et al. 2010; Moran 2012), but evidence is limited that colostrum can ever contain enough antibodies to be effective against cryptosporidiosis (Burton et al. 2011). Therefore, while we report the observations below, we suggest that this body of evidence is inconclusive about the relevance of colostrum feed to infection risk.

#### Any colostrum

Four articles assessed whether having any colostrum was effective, compared with no colostrum. Only Matoock et al. (2005) reported some colostrum to be protective (OR = 0.5, reported without variance). Only one higher quality study (Trotz-Williams et al. 2008) assessed any vs. no colostrum, finding that colostrum had no effect.

#### Suckled rather than hand- or bottle-fed colostrum

Three studies assessed whether calves suckling from dams to get colostrum was protective/risky compared with colostrum delivered via other delivery systems. Matoock et al. (2005) found bottle feeding to be more risky (OR = 3.1, no variance reported), while Trotz-Williams et al. (2007) and Silverlås et al. (2009b) found that bottle feeding had no impact on infection risk. Silverlås et al. (2009b) (which reported no effect) was the only higher quality study that assessed bottle feeding vs. suckling. Trotz-Williams et al. (2008) found that delivering colostrum by oesophageal tube (colostral drenching) did not increase risk (the alternative to colostral drenching was not specified).

#### Sterilized vs. untreated colostrum

Silverlås et al. (2009b), Weber et al. (2016) and Trotz-Williams et al. (2007) assessed whether calves had untreated (not heated, sterilized or pasteurized) colostrum. Weber et al. (2016) found that unsterilised colostrum appeared to be protective against *C. parvum* (OR 0.01, 95%CI 0–0.52), but the only higher quality study Silverlås et al. did not find that intake of unsterilised colostrum was protective.

There is weak evidence that having colostrum could be protective against *C. parvum* infection, but colostrum intake as a risk or protective factor has not been tested effectively.

There is lack of consistent evidence that any specific feeding delivery system for colostrum (or milk) is riskier or protective than others.

### Milk replacer

Two studies found that use of milk replacer (rather than real milk) led to higher risk of oocyst shedding. Díaz et al. (2018) reported higher odds of infection for calves on milk replacer (OR 3.59, 95%CI 1.2–12.2), while Trotz-Williams et al. (2008) found that use of milk replacer before age 7 days was associated with higher odds of infection (OR 1.40, 95%CI 1.06–1.85). Three other studies (Imre et al. 2015; Trotz-Williams et al. 2008; Urie et al. 2018) found that use of milk replacer made little or no difference to risk of disease. The findings of the two higher quality studies were that milk replacer either did not matter (Urie et al. (2018) or increased risk (Trotz-Williams et al. 2008). Commenting on the different findings in their own previous study (Trotz-Williams et al. 2007, which found that milk replacer was insignificant), the 2008 publication suggested that use of milk replacer might reflect other herd-level factors rather than simply effects of milk replacer. They also stated that their larger study (in 2008) had many more herds so was likely to be more reliable. Evidence that use of milk replacer increases risk is mixed and inconclusive; however, this risk factor is highly modifiable so worth exploring further.

### Herd size

Eight studies considered herd size in adjusted models, of which five did not find that herd size mattered. Three studies (Silverlås et al. 2009b; Szonyi et al. 2012; Urie et al. 2018) found higher risk of calves shedding *C. parvum* with larger herd size. The odds ratios for larger herd sizes in the adjusted models ranged from 1.55 to 292 (potentially very strong effects). All three of the highest quality studies considered herd size as a risk factor. Trotz-Williams et al. (2008) found no effect while the other two higher quality studies reported increased risk in larger herds: Silverlås et al. (2009b) (OR = 11, 95%CI 2.5–45) and Urie et al. (2018) (OR = 292, 95%CI 46–1836). There is consistent evidence that larger herds can be associated with elevated levels of *C. parvum* infection.

### History

Prior occurrence of parasitic diarrhoea (*C. parvum* or *Giardia)* on farms was considered by three studies as a risk factor for fresh *C. parvum* infection. Two studies found no support for increased risk, while Starkey et al. (2005) found that prior occurrence increased the chances that calves would shed *C. parvum* during the observation period. Silverlås et al. (2009b) is the only higher quality study that considered whether the herd had a history of relatively greater diarrhoeal disease and found that prior infections did not affect the odds of new infections. This evidence is relatively consistent, suggesting that future infections are not inevitable after initial outbreaks.

### Location attributes

Szonyi et al. (2012) addressed topographical traits including rainfall, slope, elevation and aspect. These findings are inconclusive but location attributes are not well supported as risk factors.

#### Latitude

Szonyi et al. (2012) and Al Mawly et al. (2015b) agreed that farm latitude was not linked to risk of infection. No higher quality studies tested latitude as a risk/protective factor.

#### Rainfall

Szonyi et al. (2012) found that farms with recent (previous month) precipitation of 100–150 mm were at higher risk (OR 3.35, 95%CI 1.2–9.5) than both drier and wetter farms. No other studies assessed local rainfall conditions and risk of *C. parvum* infection.

#### Slope

Szonyi et al. (2012) found that farms with average slopes (5–10% over an unclear size grid area where the farm was located) were at lower risk (OR 0.14, 95%CI 0.044–0.45) compared with farms with steeper or shallower average slopes. It seems likely this variable relates indirectly to local drainage conditions. Sischo et al. (2000) also assessed slope and run off variables on farms and did not find that either could be linked to risk of *C. parvum* infection. No higher quality studies tested slope as a risk/protective factor.

### Organic production

Two studies considered organic dairy production as a possible risk factor (vs. conventional dairy production); both studies found the risk to be higher in organic systems: Maddox-Hyttel et al. (2006) found OR 2.46 (95% CI 1.16–5.19) and Silverlås et al. (2009b) reported OR 4.9 (95% CI 1.0–15). No other studies assessed organic farming as a risk factor. Silverlås et al. is a high-quality study. There is limited but consistent evidence that organic herds have more *C. parvum*.

### Other management features

This section deals with aspects of management not described elsewhere in this summary.

#### ‘Industrial’ not ‘grazing’ management

Industrial vs. grazing management was assessed in Imre et al. (2015), who did not clearly define what they meant by these terms. In Imre et al., industrial management was associated with higher risk for *C. parvum* (OR 1.59, 95%CI 1.0–2.4). A higher quality study (Silverlås et al. 2009b) assessed something that may be similar, whether or not animals were allowed to graze outside in summer, and found that this made no difference to infection risk. There is a limited and inconclusive evidence about outside grazing or ‘industrial’ versus other management practices.

#### Isolation of sick calves from healthy calves

Matoock et al. (2005) and Silverlås et al. (2009b) both assessed aspects of quarantine: Matoock et al. considered whether having dedicated carers reduced risk of transmission in a herd, while Silverlås et al. (2009b) (a higher quality study) looked at the policy of isolating sick calves from healthy animals. Neither study found isolation to be associated with *C. parvum* infection risk.

#### Stocking density

Stocking density was assessed by Brook et al. (2008) and a higher quality study (Silverlås et al. 2009b). Evidence is limited, but no relationship between stocking density and risk of infection was found.

#### Stock rotation

Stock rotation was assessed by only one (higher quality) study (Silverlås et al. 2009b). Silverlås et al. found that farms that operated a policy that involved moving stock into areas at variable times (not all in and out at once) greatly increased risk of infection (OR 25.7, 95%CI 4.3–154). Evidence is very limited but strong in a higher quality study; this is potentially a very modifiable risk factor worth assessing further.

### Pen features

Many studies focused on aspects of stock housing which are among the most readily modifiable of risk factors.

#### Flooring

Concrete vs. other flooring options where calves are born or live has been considered often. Matoock et al. (2005) found that compared with earth flooring, concrete flooring reduced risk of *C. parvum* infection (OR 0.3, no measure of variance reported). Weber et al. (2016) found flooring type to be unimportant, but was not clear about which flooring options were compared. The higher quality study (Trotz-Williams et al. 2008) reported lower risk of disease for calves on concrete (OR 0.59, 95%CI 0.45–0.76), while other types of flooring (gravel or earth) were not associated with disease risk. They suggested this was because concrete is easier to fully clean. Related to this, and although they did not comment on flooring composition specifically, Maddox-Hyttel et al. (2006) tested for type of cleaning (pressure hosing vs. sweeping) and found this did not impact risk of disease.

The relevance of cleaning methods and potential cleanliness levels to disease risk was speculated to relate to type of flooring by Trotz-Williams et al. (2008) (concrete vs. gravel or earth). Trotz-Williams et al. (2008), a higher quality study, found that sweeping (vs. other floor cleaning such as scraping and pressure-hosing) did not stay in their models if type of flooring (e.g. concrete) was included in the model. In other words, concrete was the more predictive single element, but possible cleaning methods depended on the type of flooring; how floors were cleaned could not be separated from the type of flooring.

EU regulations on animal welfare require that calves must have a minimum depth of soft bedding (https://ec.europa.eu/food/animals/welfare/practice/farm/calves_en). There is some consistent evidence, including from a higher quality study (Trotz-Williams et al. 2008), that concrete flooring is safer than soft flooring, but this finding may relate to other hygiene practices rather than anything intrinsic to concrete as a flooring type.

Slatted flooring (that reduces animal contact with own excreta) was addressed by two studies (Díaz et al. 2018; Maddox-Hyttel et al. 2006). Díaz et al. found slatted flooring to be highly protective of *C. parvum* infection (OR 0.17, 95%CI 0.05–0.46), while Maddox-Hyttel et al. (2006) found slatted flooring not to be related to infection risk. No higher quality studies assessed risks or protection linked to slatted flooring. There is weaker quality and limited evidence that slatted flooring can reduce risk of cryptosporidiosis.

#### Types of bedding

With regard to bedding, most studies considered hay under calf quarters although Silverlås et al. (2009b) assessed bedding specifically in birthing pens. To supplement our formal findings, we mention relevant observations in Castro-Hermida et al. (2002), a study that used adjusted models but was otherwise ineligible for our review because of imprecise detection methods: Castro-Hermida et al. found that calves being housed on straw or hay (vs. bare cement) was linked to higher risk of suspected *C*. *parvum* (OR 1.6, 95%CI 1.2–2.3).

Among the studies included in this review, Szonyi et al. (2012) found that being on hay (rather than dust or no bedding) increased risk of shedding (OR 7.05, 95%CI 2.4–20.1). Four other studies (Maddox-Hyttel et al. 2006; Silverlås et al. 2009b; Urie et al. 2018; Weber et al. 2016) did not find a relationship between hay bedding and risk of disease. Of these, Urie et al. (2018) and Silverlås et al. (2009b) are higher quality studies. Urie et al. (2018) also considered other types of bedding (sand, shavings, none, combination materials) as risk factors and found no link with disease incidence. There is weak quality evidence that hay bedding may confer greater risk and no significant evidence about other types of bedding.

#### Depth of bedding

Brook et al. (2008) and Maddox-Hyttel et al. (2006) both assessed depth of bedding under calves. Maddox-Hyttel et al. found no relationship with *C. parvum* shedding. The models in Brook et al. found that disease risk was much lower when bedding was deeper (11–15 cm, OR 0.12, 95%CI 0.03–0.48), compared with shallower bedding (0–5 cm). Brook et al. tested other depths. Six to 10 cm depth was also protective compared with 0–5 cm depth (OR 0.32, 95%CI 0.11–0.95), while >15 cm depth was not protective compared with 0–5 cm depth (OR 0.72, 95%CI 0.2–2.59). So the relationship between depth and disease risk was not linear and not consistent and only tested by two lower quality studies. Evidence about optimal bedding depth is limited and inconclusive.

### Cleaning (other than floors)

Separate from flooring management decisions, seven studies considered at least one aspect of how calf housing areas were cleaned.

#### Washing feeding utensils

Díaz et al. (2018) found that use of disinfectants strongly increased risk of disease (OR 6.84, 95%CI 2.05–27.4), but did not describe exactly how the disinfectants were used. The higher quality study, Trotz-Williams et al. (2008), reported that washing feeding utensils with disinfectant (vs. soap and water or no washing) had no impact on disease risk. However, washing with soap and water (vs. no cleaning/disinfectant) did reduce infection risk (OR 0.61, 95%CI 0.46–0.82). There is limited and inconclusive evidence about the best way to clean feeding utensils.

#### Changing bedding frequency, excrement removal within calf pens

Sischo et al. (2000) reported that changing bedding >12 times a year (vs. less often) increased disease risk (OR 2.5, 95%CI 1.4–5.0). Sischo et al. surmised that this result reflected poor biosecurity, stating

The process of bedding removal involves walking and using equipment between animal groups and pens. In this process, personnel and equipment become fomites for spreading infection. A previous study found a similar effect (i.e. increased bedding management increased the probability of calves shedding) (Maldonado-Camargo et al. 1998, pp. 265).

Daily removal of bedding from the actual enclosure *was* protective in Matoock et al. (2005) (OR = 0.1, no measure of variance reported). Cleanliness aspects were not significant in four other studies, where the practices were described as follows: frequency of waste removal from pens (Maddox-Hyttel et al. 2006); cleanliness rating of bedding (Brook et al. 2008), barnyard hygiene score (Sischo et al. 2000) and general hygiene of pens (an attribute not clearly described, Díaz et al. 2018); and routines for handling manure (Sischo et al. 2000) and how many times a day that stables were cleaned (exact description not supplied) in Weber et al. (2016). Matoock et al. (2005) reported that ‘frequent’ removal of manure from the cattle enclosure areas reduced risk (‘frequent’ and ‘enclosure areas’ were not defined; OR 0.2 was reported without variance). No higher quality studies assessed cleanliness of calf bedding as a risk factor. Evidence is very weak that any specific cleaning routines consistently affect disease risk.

### Calf housing features separate from stock density, flooring or bedding

Szonyi et al. (2012) found that calves living in a cow barn (alternative unclear) greatly increased risk of disease (OR 14, 95%CI 2.5–78.8). Szonyi et al. (2012) did not link disease risk to either being in a pen or being in a greenhouse (alternatives not clearly defined). These findings may correspond to some of the data below or elsewhere in this review about similar risk factors, but the lack of clear definition of the risk factors in Szonyi et al. precludes grouping in this narrative.

#### Proximity or contact with other calves (including indirect)

The adjusted model in Maddox-Hyttel et al. (2006) found that disease risk in calves was lower if pens had an empty period (from 0 to 9 days) between new calves (OR 0.42, 95%CI 0.21–0.87). Sischo et al. (2000) found that direct contact between calves raised disease risk (OR 4.6, 95%CI 1.6–20.1). The only higher quality study that considered proximity of calves to other calves and cows was Silverlås et al. (2009b). Silverlås et al. reported that the closer calves were to other cattle, especially other calves, the higher their risk of disease. For instance, being close to other calves had OR 5.4 (1.6–19). However, the exact distance threshold used for being ‘close’ other animals is not described in Silverlås et al. There is consistent evidence (although not extensive) that more contact between young calves increases their risk of getting cryptosporidiosis.

#### Roofing

Weber et al. (2016) found that being housed in the open (no roof) greatly increased risk of disease (OR 19.9, 95%CI 2.0–199.1), while Szonyi et al. (2012) found no link between shedding oocysts and being housed outdoors or indoors. Evidence is limited and inconsistent about whether being outdoors affects risk of disease.

#### Individual boxes versus shared pens

Four studies (Brook et al. 2008; Díaz et al. 2018; Maddox-Hyttel et al. 2006; Urie et al. 2018) found that being in individual box housing (as opposed to being in a group stable) did not affect disease risk while Imre et al. (2015) reported a raised risk (OR 1.59, 95%CI 1.0–2.4) for calves housed in a shared pen. Urie et al. (2018) is the only higher quality study to look at this risk factor and found no link with disease. The balance of evidence suggests that whether calves or housed individually or in groups has no impact on disease risk.

#### Ventilation

Matoock et al. (2005) reported that calves housed in well-ventilated pens that were also exposed to sunlight had lower risk of cryptosporidiosis (OR 0.4, no variance reported). A higher quality study (Urie et al. 2018) found that natural ventilation rather than pressure tube or cross ventilation conditions had no link to disease risk. Existing evidence is mixed and limited about whether ventilation conditions affect disease risk.

#### Tied or free?

Both Szonyi et al. (2012) and Silverlås et al. (2009b) found no link between whether calves were tied (or free to roam in their enclosure) and *C. parvum* infection. Existing evidence is limited, but consistent that being tied or free in the stall does not affect disease risk.

### Co-infection

Two studies considered whether co-infection with other pathogens (known to cause bovine diarrhoea) could be linked to *C. parvum* oocyst shedding. Al Mawly et al. (2015b) concluded that both presence and severity of disease from *C. parvum* were significantly greater in the presence of co-infection with other pathogens, but did not report this sensitivity analysis in detail. Trotz-Williams et al. (2007) is the only study that reported fully if the presence of *C. parvum* infection was more likely in the presence of co-infection with another pathogen known to induce diarrhoea in bovine calves. They assessed *C. parvum*-positive status with respect to both bovine rotavirus and bovine coronavirus infections. Only a positive test for bovine coronavirus was retained in the adjusted multilevel generalized linear mixed model; co-infection with bovine coronavirus was not significantly linked to *C. parvum*-positive status at *p* < 0.05 (OR 0.59, 95%CI 0.30–1.16). Trotz-Williams et al. (2007) is a higher quality study. Evidence was consistent but limited, including from a higher quality study, that co-infection with other diarrhoea-causing pathogens makes *C. parvum* infection more likely.

### Prophylaxis (other types)

Some of the risk factor studies noted whether any animals were exposed to treatment that was meant to reduce illness, as reported below.

#### Use of halofuginone lactate or related products

Two studies mentioned that some calves were given halofuginone lactate (HfL). Díaz et al. (2018) found this strongly protective (OR 0.27, 95%CI 0.05–1.06) but Weber et al. (2016) found it had no effect. No higher quality included studies assessed HfL as risk or protective factor. Experimental evidence will better inform any assessment of efficacy of HfL. Evidence was too limited and inconsistent to assess possible benefits of HfL.

#### *Escherichia coli* or other vaccines used

Three studies assessed dams receiving an *E. coli* vaccine (e.g. Ecostar, Scourgard or none) as a risk factor. Díaz et al. (2018) found *E. coli* vaccine had no effect on cryptosporidiosis in the offspring. Trotz-Williams 2007 found the vaccine strongly protective against shedding *C. parvum* oocysts (OR 0.3, 95%CI 0.2–0.45), while the higher quality study, Trotz-Williams et al. (2008), found that the *E. coli* vaccine *increased* disease risk (adjusted OR 1.7, 95%CI 1.3–2.2). Trotz-Williams et al. (2008) also assessed another vaccine designed to prevent calf diarrhoea (First Defence, which claims to contain antibodies against bovine coronavirus and K99+ *E. coli*). First Defence was also found to *increase* incidence of cryptosporidiosis (OR 1.38, 95%CI 1.06–1.81). The increased risk may be correlative; calves receiving the vaccine may have tended to be in herds that have more history of cryptosporidiosis. Evidence in favour of *E. coli* or similar vaccines was mixed and therefore inconclusive.

#### Other coccidiostats

Trotz-Williams et al. (2007) found that coccidiostats in calf feed (specific product not specified) were protective (OR 0.67, 95%CI 0.49–0.93). Two higher quality studies (Trotz-Williams et al. 2008; Urie et al. 2018) found that use of coccidiostats in the calf diet were not protective against cryptosporidiosis. Trotz-Williams et al. (2008) specifically looked at decquinate, monensin and other unnamed coccidiostats in the feed. Urie et al. (2018) did not specify which coccidiostats were assessed. Evidence was inconsistent about whether coccidiostats were effective; higher quality studies did not find efficacy.

#### Other supplements in diet

Two higher quality studies (Trotz-Williams et al. 2008; Urie et al. 2018) found that injectable vitamin E, injected selenium, antibiotics, additives and antimicrobials in the liquid diet did not affect disease risk. Evidence was limited but consistent that other nutritional supplements did not affect risk.

#### Other preventive drugs

Urie et al. (2018) found that administering ‘preventive drugs’ was not associated with disease risk (‘preventive’ drugs were not defined). Similarly, Trotz-Williams et al. (2008) considered whether vaccination against any pathogen or ‘medications’ (also undefined) were associated with disease risk: they were not. The lack of specificity about the definitions of these posited protective factors is not unusual in this body of literature, even though they are otherwise higher quality studies.

### Season or weather

Three studies assessed risks for calves born in warmer/colder months. Starkey et al. (2005) found that being born in summer, autumn or winter was protective (OR 0.71, SE 0.35, *p* = 0.04). Trotz-Williams et al. (2007) found that being born in summer (vs. winter) was associated with higher risk of *C. parvum* infection (OR 1.58, 95%CI 1.17–2.12). One higher quality study (Urie et al. 2018), constructed a temperature–humidity index that was linked to risk of infection; calves born in higher temp/humidity months had elevated risk of infection (OR 1.01 per degree increase in °F, 0.003 SE, *p* < 0.001). Infection rates peaked in October in Urie et al. (2018) (a study that included diverse climatic zones across the USA). Most of these findings suggest that that calves born in hotter and more humid months are at higher risk of *C. parvum* infection.

## Discussion

Current livestock management strategies believed to reduce infection with *C. parvum* encompass aspects of hygiene, welfare, segregation and nutrition practices. Hygiene encompasses maintenance of rigorous cleaning and hygiene routines for both pens and animals; there are cleaning agents specifically licenced for use against *Cryptosporidium* (Morendun Foundation 2014). Deep straw bedding is thought to increase cleanliness of the animals and keep them away from faeces. Additionally, conditions should be kept as dry as possible. Disinfection (buckets or pans) should be available to staff at entrances to calf sheds. Livestock management strategies related to welfare encompass keeping animals warm and hydrated with electrolytes if necessary. Nutrition measures address whether colostrum or colostrum substitutes better bolster immune systems and overall condition (Godden 2008; Meganck et al. 2014; Wells and Thomson 2014). However, hygiene, disinfection routine, depth of bedding, segregation of sick animals and nutritional status were not revealed as important risk factors in our review. It is worthwhile to note that most studies that mentioned disinfection did not explicitly say what kind of disinfectant was used; we have stated the exact product where reported, but mostly the exact product was not reported. This omission is important because not all possible disinfectants are known to be effective against *C. parvum*.

Segregation by age groups is often advised because young animals are the most at-risk group for having illness from *C. parvum* which may be shed by older, asymptomatic animals (Wells and Thomson 2014). Our review did find evidence to support this practice (Silverlås et al. 2009b).

This study did not find any especially convincing evidence for any specific risk or protective factor. In parallel research (Brainard et al. under review; Brainard et al. 2020), we systematically reviewed all chemoprophylaxis and management strategies that have been tried in formal trials to prevent or mitigate cryptosporidiosis in young calves. Other than treatment with halofuginone, we were unable to find strong evidence in support of any specific treatment or management regime that had been subjected to experimental trial.

## Limitations

The greatest limitation in this review is power, due to limited quantity of available data. Most management practices are herd-specific. Even if a study includes 1000 animals, if it only relates to 50 herds, then for any factors related to the herd, rather than individual calves, we will have very low power to see important associations. This underpowering means that the default is that we will not see statistically significant associations. For these reasons also, we have not attempted a meta-analysis. It is very possible that our results are dismissing factors that may in reality indicate useful management strategies to reduce *C. parvum* infection.

We have restricted our synthesis to those studies that attempted multivariate analysis, to attempt to deal with potential confounding factors. This is important because management and lifestyle factors tend to cluster together, and separating out effects from these clusters is key to finding useful ways to reduce *C. parvum* infection. Nevertheless, our summary remains very reliant on imperfect author reports. Some of the observation questionnaires and data they reported may have been validated or piloted, but piloting/validation was not reported and that omission meant we could not confirm that the study was higher quality. For instance, Al Mawly et al. (2015b) describes adjusting predictions of oocyst detection using only a small number of risk factors that were collected by undescribed methods, yet we suspect these methods were subject to an undeclared verification process. On the same set of farms and animals, assessing a different outcome (risk of diarrhoeal disease from specific pathogens), Al Mawly et al. (2015a) describe extensive piloting and questionnaire development for farm-specific variables. We suspect there were many unstated risk factors that were in fact assessed in many studies. We could not find the original questionnaires used by Weber et al. (2016) or Silverlås et al. (2009b); we recognize that these authors were very thorough and may have recorded data on risk factors that were not described in reports. It is also possible that some researchers omit such extensive details for their own quality control reasons: they may realize that some observations were not made consistently. Similarly, some authors (e.g. Trotz-Williams et al. 2008) mention that they omitted comparisons for any factors which were the same for vast majority (i.e. 90%) of animals. This meant, for instance, that if 90% of calves received colostrum, it would be inappropriate to assess having colostrum as a potentially protective factor (due to too few animals in the no colostrum group for the statistical comparisons to be meaningful). Other studies may have included too few calves with *C. parvum* infections to assess any risk factors effectively.

We are acutely aware of many risk factors that were not adequately considered. Whether neonate calves had raw colostrum (not heat treated) was rarely reported. Even more difficult, it is likely that unobserved and/or unreported herd-specific factors affected the risks of a calf being ill or shedding oocysts. This missing information is a very important reason that future research needs to cluster observations by herd (not just treat each animal as individual).

A systematic review methodology for summarizing evidence is inherently conservative; this study design emphasizes only using demonstrable benefits or harms to inform policy and practice. This conservatism is meant to help prevent investment in futile measures but it cannot identify useful practices that have not been tested from types of evidence outside of the inclusion criteria. The strength of this systematic review is in highlighting how the body of evidence in risk factor studies on real animals needs to improve to make firm conclusions for better practices in herd management.

## Conclusions

The evidence base is generally insufficient to support any specific practice for controlling cryptosporidiosis in bovine calves. This is problematic because livestock managers cannot be sure which activities they should be doing to prevent this disease. Evidence-based practices are as important in veterinary science as in other biomedical sciences. Better quality and very specific evidence is needed about which modifiable risk factors should be prioritized in preventing cryptosporidiosis in calves.

No overwhelming evidence on risk or protective factors was found. The most consistent evidence was that risk of *C. parvum* infection increased when calves had more contact with other calves, were in larger herds or in organic production. Hard flooring reduced risk of infection, while calves tended to have more cryptosporidiosis during warm and wet weather. Co-infection with other pathogens was linked to being more likely to have a *C. parvum*-positive test in both studies that addressed this as a risk factor. All such factors should be formally tested in high-quality randomized controlled trials or case–control studies.

Many other risk factors were analysed but did not have consistent or conclusive effects. Being in individual or shared pens, being indoors or outdoors, whether the herd had history of cryptosporidiosis, breed, colostrum, time spent with dam after birth, type of flooring or bedding, whether calves were tied or free and use of nutritional supplements were not shown to consistently protect or increase risk of disease. However, most of these findings arose from relatively few studies: i.e. just two studies assessed each of organic production, nutritional supplements or being indoors/outdoors. Large high-quality studies across a large number of herds are needed that aim specifically to assess associations between rather than this range of management practices (including calf contact levels, herd size and organic credentials) and *C. parvum* infection. Funding for such large studies that carefully assess and report the full range of potential risk factors (to allow high-quality well-powered multivariate analysis as well as adjustment for clustering) are needed to enable the science to move forward and properly inform animal husbandry. Such studies need to use validated tools for assessment of risk factors, using pre-specified definitions, and high-quality methods of *C. parvum* detection in young calves.

## Electronic supplementary material

ESM 1 (DOCX 14 kb)
