# Supplementary material for: Systematic review of modifiable risk factors shows little evidential support for most current practices in Cryptosporidium management in bovine calves
Source: Parasitol Res. 2020 Sep 30;119(11):3571–84. doi: 10.1007/s00436-020-06890-2 (PMC7524573; doi:10.1007/s00436-020-06890-2)
Supplement: Supplementary file 1 — (DOCX 14 kb) [file 436_2020_6890_MOESM1_ESM.docx]

# Supplementary file 1

**Quality Assessment Questions** (with decision criteria)

1. Was the cohort recruited in an acceptable way?

NO, Would mean they chose a population that was in some way peculiar, unlikely to provide generalizable data for other cattle;

YES, Would mean nothing unusual about the group, or biased about the specific animals used (such as if they were non-cow crosses). *Limited Geography would not be expected to mean a NO answer*

2. Was the risk/protection exposure accurately and consistently measured to minimise bias?

YES, Reported that was some piloting of RF data collection methods, or validation of data collection

UNCLEAR, No mention of piloting or validation

NO, Clearly no piloting or validation process (they actually said no piloting/validation)

*This question is looking for any process to confirm that data collection was validated or consistent*

3. Was the CP detection consistent and valid?

*This question is about what laboratory methods they used after collection to identify the pathogen*

YES, Specific (molecular) methods that are specifically about C. parvum, consistently applied.

UNCLEAR, Not enough info or method is only microscopy but with high level of diarrhoea to be eligible, must also *say* that they think they found C. parvum or mix of Crypto species including (pathogenic) C. parvum

NO, Ineligible

4. In adjusted models: list which if any of the following confounding factors that the authors tried to adjust for (if appropriate): husbandry (which could include housing after birth, cleaning, disinfection), season, sex, duration stayed with dam, colostrum, co-infection). *However, adjustments might be appropriate if 90-95%+ of calves had same attribute, eg., were same sex, or all calves sampled on same day (no season variation).*

5. Did study take account of unknown confounding factors in multivariate models (where appropriate)? *Examples: Looking for clustering by region, herd level, farm level, or year of data collection as appropriate (eg., conditional logistical regression)*

YES, They state they clustered for at least one of these kinds of confounders or it clearly wasn’t relevant to cluster in any of those ways.

UNCLEAR, Not mentioned if clustered, or any way of reporting which is confusing

NO, Such clustering explicitly said they didn’t do

**Quality Assessment Category assignment**

Lowest bias category (dark green): YES to questions #1, #2, #3, #5 and at least 10 modifiable risk factors considered for adjusted model #4

2^nd^ lowest bias category (medium green): YES to #1, #3, #5 & at least 10 modifiable risk factors considered (#4)

2^nd^ highest bias category (light green): YES to #1, #3 and at least 5 modifiable risk factors in adj model (#4)

Highest risk category (no colour code/white): Other studies
